# Infliximab versus ciclosporin for steroid-resistant acute severe ulcerative colitis (CONSTRUCT): a mixed methods, open-label, pragmatic randomised trial

**DOI:** 10.1016/S2468-1253(16)30003-6

**Published:** 2016-09

**Authors:** John G Williams, M Fasih Alam, Laith Alrubaiy, Ian Arnott, Clare Clement, David Cohen, John N Gordon, A Barney Hawthorne, Mike Hilton, Hayley A Hutchings, Aida U Jawhari, Mirella Longo, John Mansfield, Jayne M Morgan, Frances Rapport, Anne C Seagrove, Shaji Sebastian, Ian Shaw, Simon P L Travis, Alan Watkins

**Affiliations:** a Swansea University Medical School, Swansea, UK; b College of Human and Health Sciences, Swansea University, Swansea, UK; c NHS Lothian, Western General Hospital, Edinburgh, UK; d University of South Wales, Pontypridd, UK; e Hampshire Hospitals NHS Foundation Trust, Royal Hampshire County Hospital, Winchester, UK; f Cardiff and Vale University Health Board, University Hospital of Wales, Cardiff, UK; g National Institute for Health Research (NIHR), Nottingham Digestive Diseases Biomedical Research Unit, Nottingham University Hospital NHS Trust, Nottingham, UK; h The Newcastle upon Tyne Hospitals NHS Foundation Trust, Royal Victoria Infirmary, Newcastle upon Tyne, UK; i Hull and East Yorkshire Hospitals NHS Trust, Hull, UK; j Gloucestershire Hospitals NHS Foundation Trust, Gloucester, UK; k Translational Gastroenterology Unit, Oxford University Hospitals NHS Trust, Oxford, UK

## Abstract

**Background:**

Infliximab and ciclosporin are of similar efficacy in treating acute severe ulcerative colitis, but there has been no comparative evaluation of their relative clinical effectiveness and cost-effectiveness.

**Methods:**

In this mixed methods, open-label, pragmatic randomised trial, we recruited consenting patients aged 18 years or older at 52 district general and teaching hospitals in England, Scotland, and Wales who had been admitted, unscheduled, with severe ulcerative colitis and failed to respond to intravenous hydrocortisone within about 5 days. Patients were randomly allocated (1:1) to receive either infliximab (5 mg/kg intravenous infusion given over 2 h at baseline, and again at 2 weeks and 6 weeks after the first infusion) or ciclosporin (2 mg/kg per day by continuous infusion for up to 7 days, followed by twice-daily tablets delivering 5·5 mg/kg per day for 12 weeks). Randomisation used a web-based password-protected site, with a dynamic algorithm to generate allocations on request, thus protecting against investigator preference or other subversion, while ensuring that each trial group was balanced by centre, which was the only stratification used. Local investigators and participants were aware of the treatment allocated, but the chief investigator and analysts were masked. Analysis was by treatment allocated. The primary outcome was quality-adjusted survival—ie, the area under the curve (AUC) of scores from the Crohn's and Ulcerative Colitis Questionnaire (CUCQ) completed by participants at baseline, 3 months, and 6 months, then every 6 months from 1 year to 3 years. This trial is registered with the ISRCTN Registry, number ISRCTN22663589.

**Findings:**

Between June 17, 2010, and Feb 26, 2013, 270 patients were recruited. 135 patients were allocated to the infliximab group and 135 to the ciclosporin group. 121 (90%) patients in each group were included in the analysis of the primary outcome. There was no significant difference between groups in quality-adjusted survival (mean AUC 564·0 [SD 241·9] in the infliximab group *vs* 587·0 [226·2] in the ciclosporin group; mean adjusted difference 7·9 [95% CI −22·0 to 37·8]; p=0·603). Likewise, there were no significant differences between groups in the secondary outcomes of CUCQ scores, EQ-5D, or SF-6D scores; frequency of colectomy (55 [41%] of 135 patients in the infliximab group *vs* 65 [48%] of 135 patients in the ciclosporin group; p=0·223); or mean time to colectomy (811 [95% CI 707–912] days in the infliximab group *vs* 744 [638–850] days in the ciclosporin group; p=0·251). There were no differences in serious adverse reactions (16 reactions in 14 participants receiving infliximab *vs* ten in nine patients receiving ciclosporin); serious adverse events (21 in 16 patients *vs* 25 in 17 patients); or deaths (three in the infliximab group *vs* none in the ciclosporin group).

**Interpretation:**

There was no significant difference between ciclosporin and infliximab in clinical effectiveness.

**Funding:**

NIHR Health Technology Assessment programme.

## Introduction

Ulcerative colitis is a chronic debilitating disease that affects about 150 000 people in the UK and 2 million people in Europe.^1^, ^2^ Acute severe ulcerative colitis affects up to 25% of patients, either on first presentation or later, and requires hospital admission for treatment with intravenous steroids.^3^ About 30% of these patients are resistant to steroid therapy and until 10 years ago, colectomy was the usual option.^4^, ^5^

Previous studies have proven the efficacy of both ciclosporin and infliximab in the treatment of both moderately severe steroid-resistant ulcerative colitis^6^, ^7^, ^8^ and acute, severe, steroid-resistant disease.^9^ However, their relative clinical effectiveness and cost-effectiveness are not known. We designed the CONSTRUCT trial to compare the clinical effectiveness and cost-effectiveness of infliximab and ciclosporin in the management of patients admitted unscheduled to hospital with acute severe ulcerative colitis who fail to respond to intravenous steroids.

**Research in context**

**Evidence before this study**

This trial was commissioned in 2008, at which time the efficacy of both infliximab and ciclosporin in the treatment of steroid-resistant severe colitis was known, but no rigorous comparative study had been published. A randomised trial has since reported no difference between the two drugs in short-term outcome (response to treatment, mucosal healing, colectomy rates or adverse events, assessed up to 3 months after randomisation), but there has been no comparison of clinical effectiveness or cost-effectiveness. A meta-analysis published in 2016 has reviewed the evidence from non-randomised studies that have compared the two treatments. Eight observational studies were heterogeneous in their outcome measurements and duration of follow-up, but all favoured infliximab in short-term treatment response (median follow-up 3 months) and six reported lower colectomy rates measured 3 months and 12 months after treatment was started.

**Added value of this study**

Although observational studies suggest that infliximab might be more effective than ciclosporin in the treatment of steroid-resistant severe colitis, the only randomised controlled trial previously fully reported has shown no difference in efficacy, colectomy rates, or adverse events measured up to 3 months. The CONSTRUCT trial has added to this evidence by measuring clinical effectiveness in 242 patients over 1–3 years, and has confirmed that there is no difference between the two drugs in terms of quality of life, colectomy rates, or safety.

**Implications of all the available evidence**

Although observational studies have suggested better clinical outcomes after infliximab, randomised trials indicate no advantage over ciclosporin in clinical outcomes or effect on patient quality of life. An economic evaluation done alongside our trial showed that treatment with infliximab incurs significantly greater costs to the UK National Health Service. Where resources are finite, the lower costs of treatment with ciclosporin should be considered, although with the advent of anti-TNF biosimilars, the differential is narrowing.

## Methods

### Study design and participants

We did a mixed methods, open-label, parallel-group, pragmatic randomised trial in 52 district general and teaching hospitals across England, Scotland, and Wales.^10^ Potential participants were identified after unscheduled admission with severe ulcerative colitis. Patients aged 18 years or older were recruited to the trial if they failed to respond to 2–5 days of intravenous hydrocortisone, with continuing severe disease according to Truelove and Witts' criteria^11^ or clinical judgment. All patients had either a proven histological diagnosis of ulcerative colitis, or indeterminate colitis for which clinical judgment suggested a diagnosis of ulcerative colitis rather than Crohn's disease, or symptoms typical of ulcerative colitis subsequently confirmed on histology of a colonic biopsy taken soon after admission.

We excluded patients aged younger than 18 years; from vulnerable groups (ie, with learning disability, severe mental illness or cognitive impairment, terminal illness, or prisoners) or unable to consent; with an enteric infection or histological diagnosis inconsistent with ulcerative colitis; pregnant, lactating, or fertile but unwilling to use contraception for 6 months after randomisation; with serious comorbidity, including current malignancy (except for basal cell carcinoma), immunodeficiency, recent myocardial infarction, heart failure, acute stroke, respiratory failure, renal failure, hepatic failure, or severe infection; known to be hypersensitive to infliximab, ciclosporin, or polyethoxylated oils; taking tacrolimus or rosuvastatin; needing emergency colectomy without further medical treatment; treated with either infliximab or ciclosporin in the 3 months before admission; with any other contraindication to treatment with infliximab or ciclosporin; participating in another clinical trial; or with poor English without available translation. Eligible patients were invited to participate by local investigators.

Because we anticipated difficulty in obtaining informed consent and baseline data from acutely and severely ill patients whose health was worsening, we explained the trial to patients with known or suspected acute severe ulcerative colitis as soon as possible after admission and, with consent, asked them to complete a baseline quality-of-life questionnaire. This created a pool of patients from which we recruited those who failed to respond to treatment with intravenous hydrocortisone, after further explanation and consent. The treatment of patients who did not consent to either cohort or trial was unaffected.

The protocol,^10^ patient information sheets and consent forms, all questionnaires, and amendments were approved by the Research Ethics Committee for Wales (08/MRE09/42) and local research and development committees. All patients provided written, informed consent.

### Randomisation and masking

Patient details were entered onto a web-based password-protected site (hosted by Bangor University, UK), and allocated at random to infliximab or ciclosporin. A dynamic algorithm^12^ was used to generate allocations on request, thus protecting against investigator preference or other subversion while ensuring that each trial group was balanced by centre, which was the only stratification used.

Since this was an open-label trial, local investigators and participants were aware of the treatment allocated, but the chief investigator and all analysts remained masked to allocation until the trial steering committee and data monitoring and ethics committee had reviewed and approved the analysis of the primary outcome.

### Procedures

Patients randomly allocated to receive infliximab were given 5 mg/kg by intravenous infusion over 2 h at baseline, and again at 2 weeks and 6 weeks after the first infusion, in accordance with local prescribing guidelines. Patients randomly allocated to receive ciclosporin received the drug by continuous infusion of 2 mg/kg per day, continued for up to 7 days if this resulted in improvement; then twice-daily tablets delivering 5·5 mg/kg per day, with the dose adjusted to achieve trough ciclosporin concentration of 100–200 ng/mL for 12 weeks. The drugs were dispensed by hospital pharmacies, as part of routine practice. They were not provided specifically for the trial by pharmaceutical companies, who did not support this trial in any way.

We did not mandate other therapy. Centres were encouraged to give co-trimoxazole as prophylaxis against *Pneumocystis jirovecii* pneumonia, and given discretion to start azathioprine or 6-mercaptopurine at therapeutic doses in week 4. Guidance included stopping steroids by week 12 in patients who remained well, but to restart steroids in patients who became symptomatic. After 12 weeks, all treatment was at the discretion of the patient's physician.

Quality-adjusted survival^13^ was measured as the total area under the curve (appendix p 1) described by scores from the Crohn's and Ulcerative Colitis Questionnaire (CUCQ, formerly CCQ),^14^, ^15^ which was completed by participants at baseline, 3 and 6 months, then every 6 months from 1 year to 3 years. If a participant underwent colectomy, additional questions were completed on post-operative discharge and at 4 weeks, 8 weeks, and 12 weeks, and then every 6 months. The CUCQ and its colectomy extension were developed by modifying and concurrently validating the UK Inflammatory Bowel Disease Questionnaire (UK-IBDQ)^16^ to be appropriate for use by patients across a range of disease states, including quiescent, mild chronic, and acute severe colitis, and post-colectomy. Although by convention low scores indicate better health on disease-specific patient-reported outcome measures, for the purposes of presenting the area under the curve, the CUCQ score was transformed so that a lower score indicated worse health. Data for the generic quality-of-life measures SF-12 (from which SF-6D was derived) and EQ-5D were collected at the same timepoints as the CUCQ.

This was a mixed methods trial that also evaluated cost-effectiveness through a cost utility study done alongside the trial. The methods and results of the cost-effectiveness study are available in detail elsewhere.^14^ In summary, costs were assessed by prospectively monitoring total health service resource use by patients in both groups of the trial, collected in case report forms at each follow-up point. These data were multiplied by relevant unit costs and expressed in 2012–13 prices. Effectiveness was assessed in terms of quality-adjusted life-years generated from EQ-5D data. We also sought the views of patients and professionals during this mixed methods study. The method and findings are reported in full elsewhere^14^ and summarised in the appendix (pp 4–5).

### Outcomes

The primary outcome measure was quality-adjusted survival^13^ because we wished to compare the effectiveness of treatment as perceived by patients over at least a year, during which time they might experience many different health states including a post-colectomy stoma.

Secondary outcome measures included change in CUCQ and change in two generic quality-of-life measures (SF-12, EQ-5D). Case report forms were completed by local research professionals, and from these were derived additional secondary outcomes of mortality; incidence of colectomy, both emergency and planned; and length of stay.

Adverse events were monitored via reports from principal investigators, and used to identify the incidence of malignancies, serious infections, and renal disorders. All incident malignancies were classified as being possibly related to the treatment received. Because of differing pharmacokinetics, we classified infections as possibly related if the diagnosis was within 1 month of the last dose of ciclosporin, or 6 months after infliximab. Thereafter infections were classified as unlikely to be related. New symptoms arising after treatment were documented in adverse event reports, and analysed by clinical system affected when severe enough to be associated with prolongation of hospital stay or readmission. Readmissions for any reason were noted in case report forms and patient follow-up questionnaires, and included in the analysis of cost-effectiveness.^15^ We had also intended to record incidence of new symptoms during treatment, or attributable to treatment, as secondary outcomes; however, completeness of case report forms was not optimal, and thus we were unable to analyse this endpoint.

### Statistical analysis

Our hypothesis was that there is no difference in the clinical effectiveness of these two treatments, as measured by quality of life. Our original target sample size was 360 participants with analysable data, based on an equivalence design, an effect size of 0·30, and a primary outcome of change in CUCQ scores at 2 years. In 2012, slower recruitment than predicted led us to reduce the analysable sample size to 250, still sufficient to detect an effect size of 0·35 with 80% power at 5% significance level. To mitigate the effect of attrition we introduced a length of follow-up of 1–3 years, and redefined our primary outcome as quality-adjusted survival, measured as the area under the curve described by CUCQ scores (including after colectomy).

Our primary analysis was by treatment allocated, reflecting the pragmatic nature of the trial design. The primary outcome measure used a general linear model to estimate differences in quality-adjusted survival between groups, adjusting for covariates including trial site, age, sex, ethnic group, social deprivation (derived from truncated post codes), baseline quality of life, disease severity, and time in follow-up.

Secondary analyses adjusted for the same covariates as the primary analysis and compared between groups: quality-adjusted survival per day (again using general linear models); CUCQ scores (using methods for repeated measures); proportion of participants undergoing colectomy (using binary logistic regression); time to colectomy (censored at the end of follow-up, and analysed by Cox regression); proportion of participants with one or more adverse events (using binary logistic regression); and mortality.

Residual diagnostics were examined in analyses that assume normality, with the options of data transformation and bootstrapping when residual distributions were markedly non-normal. Identified outliers were excluded and the revised datasets reanalysed. Analyses are summarised by descriptive comparisons between groups in accordance with CONSORT guidelines,^17^ notably estimates with 95% CI representing two-tailed tests at the 5% significance level.

The study was overseen by a data monitoring and ethics committee. Statistical analyses were done with SPSS version 22. This study is registered with the ISRCTN Registry, number ISRCTN22663589.

### Role of the funding source

The study funder had no role in the design of the study apart from the detailed scrutiny and feedback from their independent peer reviewers before funding was awarded. The trial was sponsored by Swansea University, whose clinical trials unit contributed to the design of the study, analysis, and reporting of the data through AW and HAH. AW, HAH, DC, MFA, ML, JMM, and JGW had access to the raw data, but remained masked to allocations until the analysis of the primary outcome had been approved at a joint meeting of the trial steering committee and data monitoring committee. All authors reviewed the manuscript and JGW had final responsibility for the decision to submit for publication.

## Results

**Figure 1:**
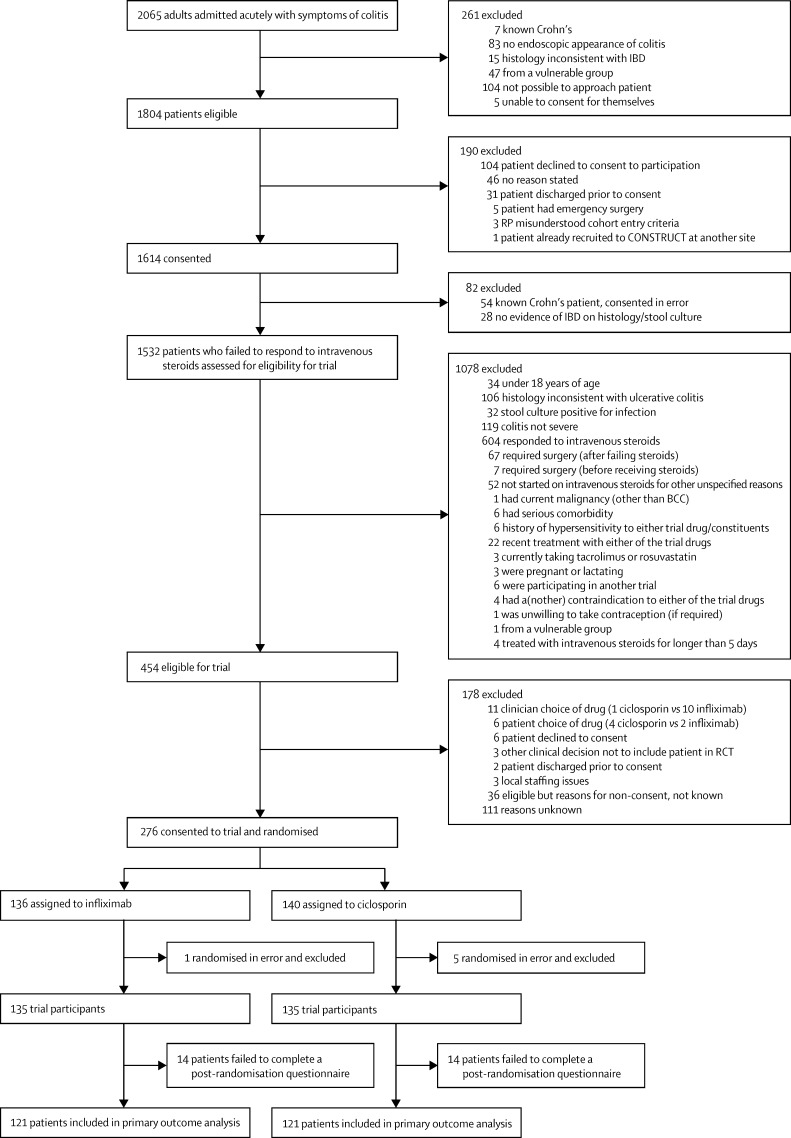
Patients included in primary outcome analysis BCC=basal-cell carcinoma. IBD=inflammatory bowel disease. RP=research professional. RCT=randomised controlled trial.

**Figure 2:**
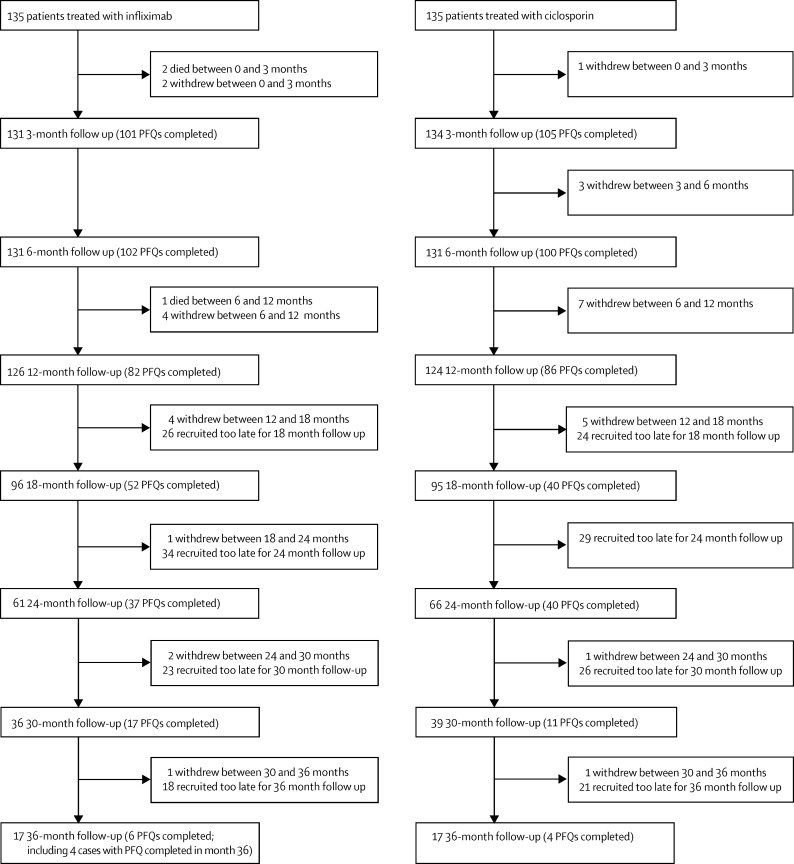
Patients included in follow-up PFQ=participant follow-up questionnaire.

Between May 25, 2010, and Feb 28, 2013, 2065 potentially eligible patients were admitted to 62 hospitals in England, Scotland, and Wales, of whom 1614 consented to inclusion in the study. From June 17, 2010, to Feb 26, 2013, 270 of these patients were recruited into the trial at 52 hospitals, and were followed up for 1–3 years until Feb 28, 2014. 135 patients were randomly assigned to each treatment group (figure 1), of whom 242 (90%) contributed to definitive analysis of the primary outcome (figure 2). The remaining 28 participants failed to complete a post-randomisation questionnaire, although any data relating to secondary outcomes were analysed.

Median follow-up was 765 days (IQR 569–966) overall (766 days [563–967] for the infliximab group and 764 days [569–954] for the ciclosporin group). At baseline, there were no significant differences between the groups in demographic or disease characteristics, haemoglobin concentration, inflammatory markers, albumin levels, or quality-of-life scores (table 1). The duration of treatment with intravenous steroids was similar in both groups (mean 5·32 days [SD 2·66] before infliximab *vs* 5·43 days [2·89] before ciclosporin). Failure to respond was assessed by clinical judgment rather than Truelove and Witts' scores in 36 patients in both groups.

There was no significant difference in quality-adjusted survival between infliximab and ciclosporin: the observed mean total area under the CUCQ curve was 564·0 (SD 241·9) in the infliximab group and 587·0 (226·2) in the ciclosporin group (mean adjusted difference 7·9 [95% CI −22·0 to 37·8]; p=0·603). The observed mean area under the curve (AUC) per day was 0·705 (SD 0·181) in the infliximab group and 0·733 (0·158) in the ciclosporin group (mean adjusted difference 0·030 [95% CI −0·009 to 0·068]; p=0·129).

**Figure 3:**
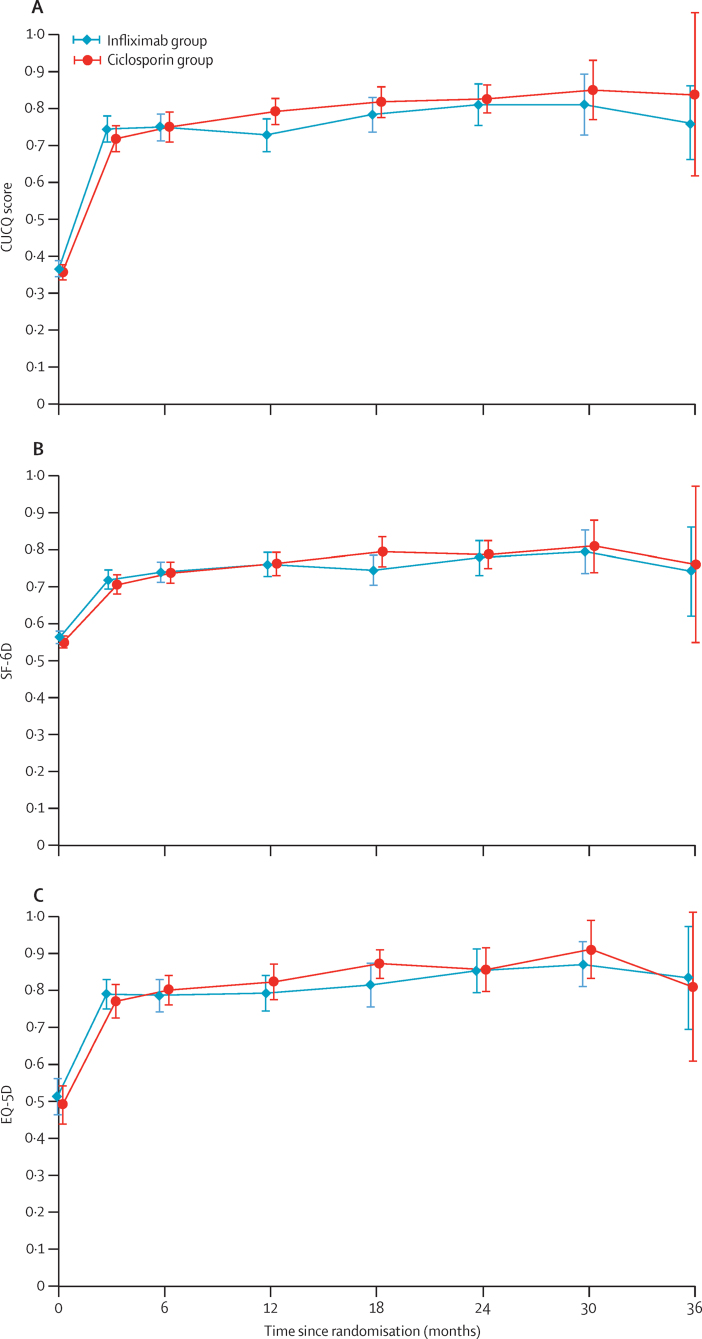
CUCQ score (A), SF-6D score (B), and EQ-5D score (C) over time Data are mean score (95% CI) for each group.

**Figure 4:**
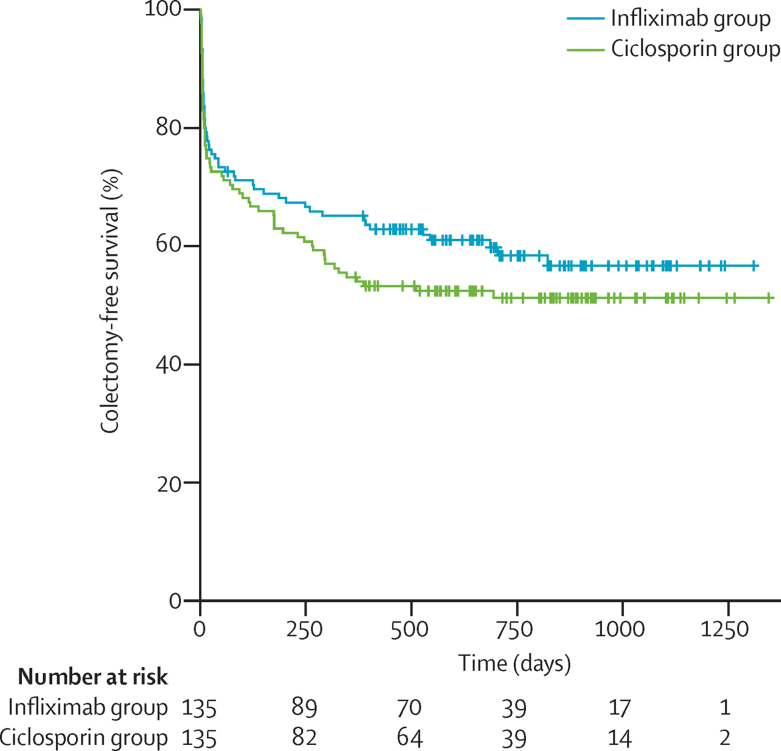
Time to colectomy

At no time after randomisation was there any significant difference between allocated groups for the secondary outcomes (appendix p 2). There was no significant difference between groups in terms of CUCQ scores (mean adjusted difference in AUC/day of survivors 0·020 [95% CI −0·019 to 0·0581]; p=0·319; figure 3A), SF-6D scores (mean adjusted difference 0·005 [95% CI −0·025 to 0·035]; p=0·737; figure 3B), or EQ-5D scores (QALY mean adjusted difference 0·021 [95% CI −0·032 to 0·096]; p=0·350; figure 3C). Furthermore, there was also no significant difference between allocated groups in colectomy rates (in-hospital: 29 [21%] of 135 patients in the infliximab group *vs* 34 [25%] of 135 patients in the ciclosporin group; at 3 months: 39 [29%] *vs* 41 [30%]; 12 months: 47 [35%] *vs* 61 [45%]; overall: 55 [41%] *vs* 65 [48%]; odds ratio [OR] 1·350 [95% CI 0·832 to 2·188]; p=0·223); or time to colectomy (mean time to colectomy 811 [95% CI 707–912] days in the infliximab group *vs* 744 [638–850] days in the ciclosporin group; hazard ratio 1·234 [95% CI 0·862 to 1·768]; p=0·251; figure 4). Although length of stay after randomisation ostensibly did not differ between allocated groups (mean 10·32 [SD 13·55] in the infliximab group *vs* 12·21 [10·18] in the ciclosporin group; mean adjusted difference 1·542 days [95% CI −1·297 to 4·381], assuming normal distribution of residuals in general linear model; p=0·286), the distribution was so skewed as to invalidate the assumption of normality. We therefore log transformed length of stay data, and found that stay after first dose of ciclosporin was significantly longer than after the first dose of infliximab (by a multiplicative factor of 1·523 [95% CI 1·278 to 1·817]; p<0·0001; appendix p 2).

**Figure 5:**
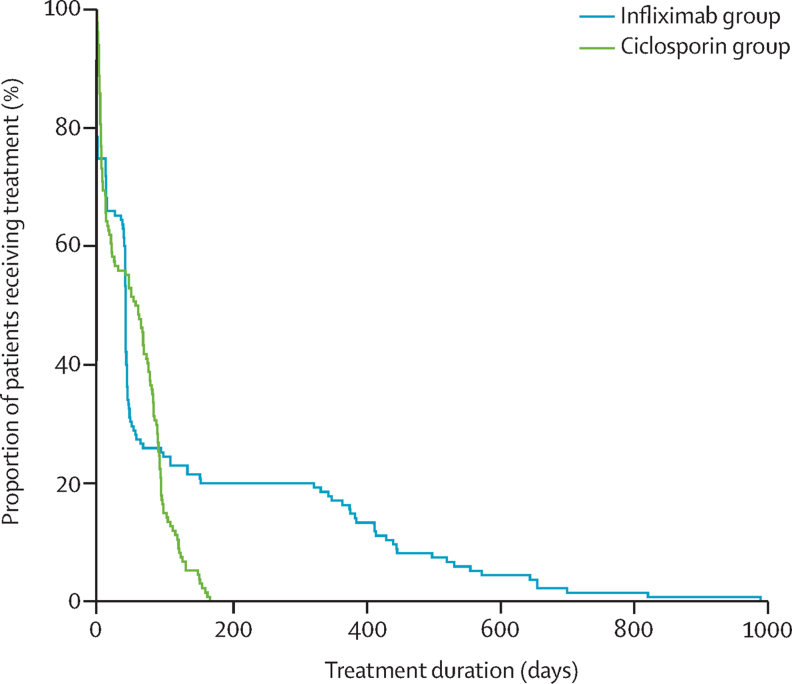
Duration of treatment

Treatment with infliximab was continued for longer than ciclosporin after the designated intervention period (figure 5); no patient received ciclosporin after 6 months, but many participants continued to receive infliximab for 2 years or more, resulting in mean treatment durations of 126 (SD 202) days for infliximab versus 56 (48) days for ciclosporin. Median treatment duration was 43 (IQR 1–99) days for infliximab versus 60 (7–93) days for ciclosporin. Nine participants assigned to ciclosporin were subsequently given infliximab (four at 3 months; two at 6 months; and three at 12 months after randomisation). One participant randomly assigned to infliximab received oral ciclosporin at 3 months. There were no significant differences between the two groups in use of azathioprine, 6-mercaptopurine, or methotrexate at any timepoint (table 2), either when given alone or in combination.

There was no significant difference between the two drugs in serious adverse reactions, or serious adverse events: 16 serious adverse reactions were noted in 14 participants given infliximab and ten in nine given ciclosporin (event ratio 0·938 [95% CI 0·590–1·493]; p=0·788; table 3). 21 serious adverse events (not related to disease progression or colectomy) were noted in 16 participants given infliximab and 25 in 17 patients given ciclosporin (event ratio 1·075 [95% CI 0·603–1·917]; p=0·807). Table 3 shows the clinical systems affected for serious adverse reactions. There were two malignancies noted in the infliximab group (basal cell carcinoma and colorectal cancer), and one in the ciclosporin group (endometrial cancer). 11 participants were noted to have impaired renal function on ciclosporin but only one was reported as a serious adverse reaction, and all resolved with dose reduction. More infections were attributed to infliximab (eight [6%] in 135 patients) than ciclosporin (one [<1%] of 135 patients), but more serious adverse events due to an infection unrelated to the intervention occurred after ciclosporin (16 [12%]) than infliximab (eight [6%]). Three patients died, all after taking infliximab: two of perioperative pneumonia with sepsis (at 20 days and 65 days after start of treatment; both had multiple comorbidities including diabetes); and one of disseminated colorectal cancer (at 278 days, 20 years after ulcerative colitis was first diagnosed).

The cost-utility analysis found that total health service costs over 30 months were £5632 (95% CI 2773–8305) higher for patients receiving infliximab, mainly due to the higher acquisition costs for infliximab (p=0·001). Effectiveness over this period was similar in both groups, and the mean adjusted difference in quality-adjusted life-years was not significant (0·021 [95% CI −0·032 to 0·096]; p=0·350).

## Discussion

This study has shown that, although both infliximab and ciclosporin improve quality of life in patients with acute severe ulcerative colitis that has not responded to intravenous steroids, there was no significant difference in quality of life between groups. Further, 40% of patients still underwent colectomy within a year (with no difference noted between groups in colectomy rate); there were also no significant differences between allocated treatments in adverse events or mortality, although three patients died of complications that were possibly related to infliximab (two sepsis and one cancer). Despite more infections possibly related to treatment being noted in patients treated with infliximab than in those given ciclosporin, participants in the infliximab group were treated with the drug for longer, and differing pharmacokinetics were also taken into account when assessing the length of time during which relatedness was possible.

Our trial was pragmatic, done in 52 hospitals, and designed to reflect current clinical practice across the UK. Local investigators were aware of treatment allocations, but the chief investigator and analysts remained masked to allocations until the trial steering and data monitoring committees had approved the analysis of the primary outcome. The protocol mandated either three infusions of infliximab over 6 weeks, or intravenous ciclosporin for up to 7 days, followed by oral administration for 12 weeks. After this, principal investigators were given discretion to continue or stop treatment. In keeping with current practice, infliximab tended to be used for longer than ciclosporin. Although increased treatment duration might have improved the effectiveness of infliximab, it certainly increased costs. Treatment with immunosuppressants during and after infliximab or ciclosporin was similar in both groups. Neither the rate nor timing of colectomy differed between allocated groups. Importantly, post-colectomy quality-of-life scores and interviews with participants who had undergone surgery both suggest that colectomy is not a bad outcome. There is evidence from observational studies that the cumulative rate of colectomy increases over time, not only with ciclosporin, but also with infliximab.^18^, ^19^, ^20^, ^21^, ^22^, ^23^, ^24^ Hence, we plan to follow the trial and cohort participants for 10 years from recruitment, using routine National Health Service data to monitor readmissions and colectomies, with annual questionnaires to monitor trial patients' quality of life.

To measure effectiveness from a patient perspective we used the CUCQ, a 32 item questionnaire with an additional ten questions for patients with a stoma. This patient-reported outcome measure was derived from the UK-IBDQ,^16^ and validated concurrently during the trial.^14^ We used it to assess quality of life of participants for 1–3 years as they passed through different health states, including colectomy and stoma. The concept of quality-adjusted survival is not new,^13^ but it is—to the best of our knowledge—the first time it has been applied to inflammatory bowel disease.

Our findings of equivalent effectiveness reinforce the efficacy findings of CySIF,^9^ a European trial that assessed treatment failure at 3 months as the primary outcome. 3-year follow-up data from CySIF reported in oral presentation in 2015 do not show differences in colectomy rate, even though most patients continued infliximab and many allocated to ciclosporin subsequently switched to infliximab.^25^ The findings of these two trials show that there is no difference between infliximab and ciclosporin in clinical efficacy or effectiveness in the treatment of steroid-resistant acute severe ulcerative colitis, in contrast with the conclusions of eight non-randomised studies, which suggested better outcomes with infliximab.^26^

The cost-utility analysis found that UK National Health Service costs over 30 months were £5632 higher for patients treated with infliximab, due mainly to the higher acquisition costs for infliximab. On this basis, ciclosporin is the more cost-effective treatment in the UK, although differences in the cost of ciclosporin and infliximab are apparent worldwide. With the advent of anti-TNF biosimilars, the cost of infliximab is falling.^27^ Nevertheless, while the cost remains higher than ciclosporin, our findings question the justification of treating patients with infliximab since it does not produce any additional health benefits. Although we accept that economic grounds are not the only grounds for decision making, the opportunity cost to other patients has to be borne in mind when choosing a treatment option that is not cost-effective.

We note that the US Food and Drug Administration has expressed its dissatisfaction with current use of disease activity scores as primary endpoints in inflammatory bowel disease trials, and is moving towards patient-reported outcome measures.^28^ This study is—to the best of our knowledge—the first major pragmatic drug trial in inflammatory bowel disease to use a disease-specific patient-reported outcome measure to assess primary outcome, and used an instrument that enabled measurement of change in quality of life through different disease states, including after surgery. This will provide a benchmark for the evaluation of re-costed infliximab, and a model to assess newer biological treatments or colonic release preparations of ciclosporin.^29^ We hope our innovative approach will also be a model for inflammatory bowel disease trials in the future.

## Figures and Tables

**Table 1:** Baseline demographic and clinical characteristics

		**Infliximab group (n=135)**	**Ciclosporin group (n=135)**
Age at randomisation (years)		35 (27–50)	36 (27–50)
Sex			
	Female	46/135 (34%)	54/135 (40%)
	Male	89/135 (66%)	81/135 (60%)
Ethnic origin			
	White	126/134 (94%)	124/133 (93%)
	Asian or Asian British	5/134 (4%)	7/133 (5%)
	Black or Black British	2/134 (1%)	1/133 (<1%)
	Other	1/134 (<1%)	1/133 (<1%)
Weight (kg)		72·9 (64·4–82·5)	70·9 (64·6–84·2)*
Smoking status			
	Never or history unknown	58/130 (45%)	75/134 (56%)
	Current or ex-smoker	72/130 (55%)	59/134 (44%)
Family history			
	Yes (any one of mother, father, sibling, or child)	28/132 (21%)	19/135 (14%)
	No	104/132 (79%)	116/135 (86%)
Condition severity (Truelove and Witts' criteria)			
	Severe	97/133 (73%)	95/131 (73%)
	Not severe	36/133 (27%)	36/131 (27%)
Mayo score			
	0	2/131 (2%)	1/128 (<1%)
	1	2/131 (2%)	2/128 (2%)
	2	35/131 (27%)	35/128 (27%)
	3	92/131 (70%)	90/128 (70%)
Montreal score			
	E1	7/124 (6%)	10/126 (8%)
	E2	64/124 (52%)	54/126 (43%)
	E3	53/124 (43%)	62/126 (49%)
Haemoglobin (g/dL)		12·7 (11·1–14·0)*	12·6 (10·7–13·5)*
C-reactive protein (mg/dL)		62·0 (21·5–128·5)†	54·0 (22·8–106·1)*
Albumin (g/L)		33 (29–37)‡	33 (28–38)‡
Receiving azathioprine, 6-mercaptopurine, or methotrexate at baseline			
	At least one	16/135 (12%)	26/135 (19%)
	None	119/135 (88%)	109/135 (81%)
Duration of symptoms for current episode (days)		21 (14–42)§	28 (14–42)¶
Duration of intravenous hydrocortisone (days)		5·0 (3·3–6·0)‖	5·0 (4·0–6·0)**
	Mean EQ-5D	0·519 (SD 0·296)††	0·496 (SD 0·314)†
	Mean CUCQ	0·366 (SD 0·133)*	0·357 (SD 0·133)†

Data are median (IQR), n (%), or n/N (%), unless otherwise indicated.

* n=134.

† n=133.

‡ n=130.

§ n=135.

¶ n=131.

‖ n=108.

** n=110.

†† n=132.

**Table 2:** Number of patients on immunosuppressants (thiopurines or methotrexate) at each time period

	**Infliximab group**	**Ciclosporin group**
Pre-baseline	16/135 (12%)	26/135 (19%)
3 months	56/131 (43%)	66/134 (49%)
6 months	56/131 (43%)	57/131 (44%)
12 months	39/126 (31%)	45/124 (36%)
18 months	23/96 (24%)	19/95 (20%)
24 months	18/61 (30%)	20/66 (30%)
30 months	10/36 (28%)	9/39 (23%)
36 months	4/17 (24%)	3/17 (18%)

Data are n/N (%).

**Table 3:** Summary of adverse events and clinical system affected in serious adverse reactions

			**Infliximab group**	**Ciclosporin group**
SUSAR			0	0
Serious adverse reaction			16	10
Total serious adverse events			145	178
	IBD related		36	47
	Surgery related		88	106
	Other		21	25
Adverse reaction			48	75
Adverse event			91	91
Serious adverse reactions by clinical system				
	Infection		8	1
		*Clostridium difficile*	1	0
		Chest infection	3	0
		Skin infection	0	1
		Post-surgical	1	0
		Other	3	0
	Neurological		2	3
	Gastrointestinal		1	2
	Renal		0	2
	Malignancy*		1	1
	Allergy or infusion reaction		2	0
	Psychiatric		1	0
	Respiratory		1	0
	Hepatic		0	1
	Other		0	0

SUSAR=suspected unexpected serious adverse reaction. IBD=inflammatory bowel diease.

* One participant on infliximab developed a cutaneous basal cell carcinoma, but was not admitted for treatment, and the event was therefore classified as an adverse reaction. There were therefore two malignancies in the infliximab group (the basal cell carcinoma and colorectal cancer), and one in the ciclosporin group (endometrial cancer). The colorectal cancer in the infliximab group was unusual in that it had some histological features suggestive of a lung primary. The local multidisciplinary team considered the case carefully and concluded that this was a single colorectal primary.
