# Supplementary material for: Infliximab versus ciclosporin for steroid-resistant acute severe ulcerative colitis (CONSTRUCT): a mixed methods, open-label, pragmatic randomised trial
Source: Lancet Gastroenterol Hepatol. 2016 Sep;1(1):15–24. doi: 10.1016/S2468-1253(16)30003-6 (PMC4994668; doi:10.1016/S2468-1253(16)30003-6)
Supplement: Supplementary appendix [file mmc1.pdf]

THE LANCET
Gastroenterology & Hepatology

Supplementary appendix

This appendix formed part of the original submission and has been peer reviewed. We post it as supplied by the authors.

Supplement to: Williams JG, Alam MF, Alrubaiy L, et al, for the CONSTRUCT investigators. Infliximab versus ciclosporin for steroid-resistant acute severe ulcerative colitis (CONSTRUCT): a mixed methods, open-label, pragmatic randomised trial. *Lancet Gastroenterol Hepatol* 2016; published online June 22. http://dx.doi.org/10.1016/S2468-1253(16)30003-6.

Web Appendix

Infliximab versus ciclosporin for steroid-resistant acute severe ulcerative colitis (CONSTRUCT): a mixed methods, open-label, pragmatic randomised trial

Method for measurement of quality adjusted survival

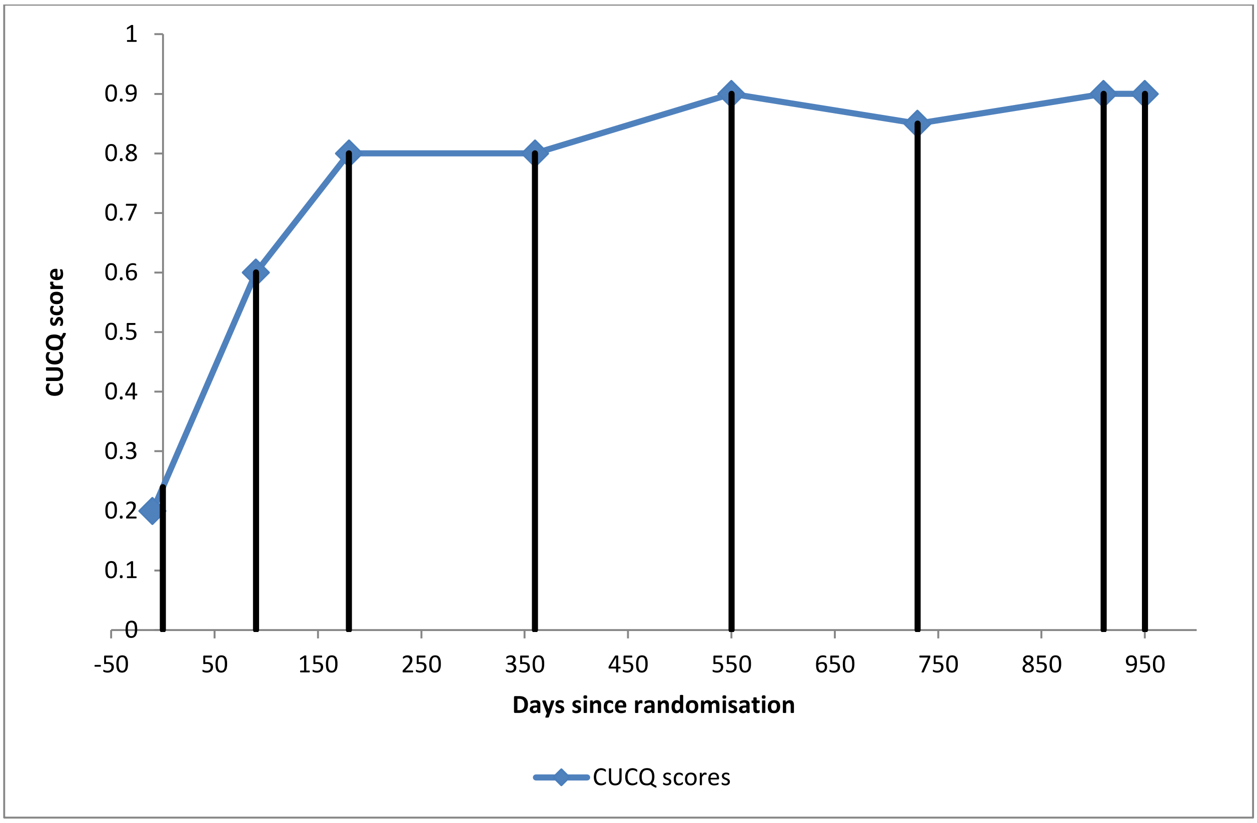

Primary and safety outcomes, analysed by treatment allocated

	Raw data		Adjusted comparison	95% CI	Intra-cluster correlation	
Outcome	Infliximab	Ciclosporin			Estimate	95% CI
QAS [note 1]:<br>Mean<br>(sd)<br>[n]	564.0<br>(241.9)<br>[121]	587.0<br>(226.2)<br>[121]	Δ=7.90<br>(p=0.603)	(-21.97, 37.77)	0.065	(0.015, 0.147)
QAS per day [note 2]:<br>Mean<br>(sd)<br>[n]	0.705<br>(0.181)<br>[121]	0.733<br>(0.158)<br>[121]	Δ=0.030<br>(p=0.129)	(-0.009, 0.068)	0.094	(0.028, 0.189)
Participants subsequently undergoing colectomy: proportion (%)	55/135 (40.7%)	65/135 (48.1%)	OR=1.350<br>(p=0.223)	(0.832, 2.188)	0	n/a
Time to colectomy (days) [note 3]:<br>Mean<br>[n]	810.8<br>[135]	744.1<br>[135]	HR=1.234<br>(p=0.251)	(0.862, 1.768)	0	n/a
Total number of SARs<br>One SAR per participant<br>Two SARs per participant	16<br>12<br>2	10<br>8<br>1	ER=0.938<br>(p=0.788)	(0.590, 1.493)	0	n/a
Participants with one or more SARs [note 4]: proportion (%)	14/135 (10.4%)	9/135 (6.7%)	OR=0.660<br>(p=0.338)	(0.282, 1.546)	0.050	(0.008, 0.132)
Total number of SAEs<br>One SAE per participant<br>Two SAEs per participant<br>Three SAEs per participant<br>Four SAEs per participant	21<br>12<br>3<br>1<br>0	25<br>13<br>2<br>0<br>2	ER=1.075<br>(p=0.807)	(0.603, 1.917)	0	n/a
Participants with one or more SAEs [note 5]: proportion (%)	16/135 (11.9%)	17/135 (12.6%)	OR=0.999<br>(p=0.998)	(0.473, 2.114)	0	n/a
Post-randomisation LOS (days) [note 6]:<br>Mean<br>(sd)<br>[n]	10.32<br>(13.55)<br>[135]	12.21<br>(10.18)<br>[135]	Δ=1.542<br>(p=0.286)	(-1.297, 4.381)	0.025	(0.002, 0.089)
Logarithm of Post-randomisation LOS [note 7]:<br>Mean<br>(sd)<br>[n]	1.878<br>(0.887)<br>[135]	2.289<br>(0.626)<br>[135]	Δ=0.421<br>(p<0.0001)	(0.245, 0.597)	0.024	(0.001, 0.085)
Mortality:<br>Proportion (%)	3/135 (2.2%)	0/135 (0%)				

ER, event ratio; HR, hazard ratio; LOS, length of stay; n/a, not applicable

Significant covariates and factors:

1. Days in follow-up (p< 0.001); CUCQ at baseline (p < 0.001); EQ-5D at baseline (p = 0.015)
2. CUCQ at baseline (p< 0.001); weight (p = 0.011)
3. Intracluster correlation assessed using time to event
4. Age at randomisation (p= 0.006)
5. Age at randomisation (p= 0.031); symptoms duration (p = 0.049)
6. Age at randomisation (p < 0.001); gender (p = 0.034); smoking (p = 0.032)
7. Age at randomisation (p < 0.001); gender (p = 0.013); EQ-5D at baseline (p= 0.007)

Sites and Principal Investigators

Trust/Health Board	Principal Investigator
Abertawe Bro Morgannwg Uni Health Board	Dr Linzi Thomas
Aneurin Bevan University Health Board	Dr Vivek Goel
Barking Havering and Redbridge University Hospitals NHS Trust	Dr Stephen Grainger
Barts Health NHS Trust	Dr James Lindsay
Blackpool Teaching Hospitals NHS Foundation Trust	Dr Peter Isaacs
Bradford Teaching Hospitals NHS Foundation Trust	Dr Conrad Beckett
Brighton & Sussex University Hospitals NHS Trust	Dr Alan Ireland
Cardiff & Vale University Health Board	Dr Barney Hawthorne
Chelsea & Westminster NHS Foundation Trust	Dr Alan Steel
Chesterfield Royal Hospital NHS Foundation Trust	Dr David Elphick
City Hospitals Sunderland NHS Foundation Trust	Dr David Hobday
Colchester Hospital University NHS Foundation Trust	Dr Achuth Shenoy
Countess of Chester Hospital NHS Foundation Trust	Dr Carol Francis
Derby Hospitals NHS Foundation Trust	Dr Andy Cole
Dorset County Hospitals NHS Foundation Trust	Dr Chris Hovell
Frimley Park Hospital NHS Foundation Trust	Dr Sarah Langlands
Gateshead Health NHS Foundation Trust	Dr Jamie Barbour
Gloucestershire Hospitals NHS Foundation Trust	Dr Ian Shaw
Hampshire Hospitals NHS Foundation Trust	Dr John Gordon
Hull & East Yorkshire NHS Trust	Dr Shaji Sebastian
Leeds Teaching Hospitals NHS Trust	Dr Alex Ford
Lewisham & Greenwich NHS Trust	Dr John O'Donohue
Maidstone & Tunbridge Wells NHS Trust	Dr Bijay Baburajan
NHS Forth Valley	Dr David Watts
NHS Highland	Dr Lindsay Potts
NHS Lothian	Dr Ian Arnott
North Cumbria University Hospitals NHS Trust	Dr Chris Macdonald
North Tees & Hartlepool NHS Foundation Trust	Dr Matt Rutter
Nottingham University Hospitals NHS Foundation Trust	Dr Aida Jawhari
Oxford Radcliffe Hospitals NHS Trust	Professor Simon Travis
Rotherham NHS Foundation Trust	Dr P Basumani
Royal Bournemouth & Christchurch Hosp NHS Foundation Trust	Dr Sean Weaver
Royal Devon & Exeter Foundation Trust	Dr Tariq Ahmad
Royal Liverpool & Broadgreen University Hospitals NHS Trust	Dr Keith Leiper* & Dr S Subramanian
Royal Shrewsbury & Telford NHS Trust	Dr Mark Smith
Royal Wolverhampton NHS Trust	Dr Brian McKaig
Salford Royal NHS Foundation Trust	Dr Andrew Robinson
Sheffield Teaching Hospitals NHS Foundation Trust	Dr Alan Lobo
South Devon Healthcare NHS Foundation Trust	Dr Cathryn Edwards
South Tees Hospitals NHS Foundation Trust	Dr Helen Dallal & Dr A Ramadas
South Tyneside NHS Foundation Trust	Dr Faheem Butt
St George's University of London	Dr Richard Pollok
Taunton & Somerset NHS Foundation Trust	Dr Paul Thomas
The Newcastle upon Tyne Hospitals NHS Foundation Trust	Dr John Mansfield
University College London Hospitals NHS Foundation Trust	Dr Stuart Bloom
University Hospital South Manchester NHS Foundation Trust	Dr Gill Watts
University Hospital Southampton NHS Trust	Dr Fraser Cummings
University Hospitals Bristol NHS Foundation Trust	Dr Tom Creed
West Middlesex University Hospital NHS Trust	Dr Joel Mawdsley
Western Sussex Hospitals NHS Trust	Dr Andy Li
Weston Area Health NHS Trust	Dr Alex di Mambro & Dr David Parker
Wrightington, Wigan and Leigh NHS Foundation Trust	Dr Yeng Ang & Dr Neeraj Prasad
* deceased	

Views of Patients and Professionals

The following is a summary of the qualitative elements of the CONSTRUCT trial. They are reported in depth elsewhere.[1]

Aim

The qualitative components of the CONSTRUCT trial used interviews to explore the views of participating patients, doctors and nurses about severe ulcerative colitis and its management. The aims were to clarify participants' feelings about their condition, their perceptions of treatment with infliximab, ciclosporin or surgery and to understand changes to these views over time. Interviews with doctors and nurses aimed to understand their views about the efficacy, safety and administration of the trial drugs, shared decision-making with patients, and their responses to their patients' ongoing illness experience.

Method

Trial participants who indicated their willingness to be interviewed when giving consent were chosen according to a purposive quota sampling framework. This identified 12 consenting patients from each arm of the trial, who were interviewed twice, two to three, and 8-12 months after randomisation. All interviews followed a semi-structured format to ensure consistency of data collection whilst enabling patients to respond to prompts if they so wished, to ensure comprehensive and rich data capture. The first interviews investigated patients' priorities for their health and well-being, and the administration, side effects and response to the treatment they received. The second interviews used a similar schedule, but included additional questions examining what had happened to them following treatment, including changes over time in their opinions of treatment, their interactions with healthcare professionals, and their current health. Patient interviews were undertaken face-to-face or by telephone depending on patient preference.

Principal investigators and nurses responsible for administering and monitoring the trial drugs were sampled purposively from trial sites based on recruitment rates to the cohort and trial. They were approached by telephone or email, and gave informed consent. All interviews were semi-structured and undertaken by telephone.

All interviews were recorded and transcribed. Patient and professional data were analysed using schema and thematic analysis frameworks that were refined over time by researchers with expertise in qualitative data. Transcripts were coded to reveal major and minor themes and categories and were also schematised to disclose succinct, multi-disciplinary overviews of key issues arising.[2-4]

Results – interviews with participants

Thirty-five interviews were undertaken with 20 participants, 15 of whom were interviewed on two occasions. The participants were split evenly between the infliximab and ciclosporin arms of the trial, and were representative of the main study population in all baseline characteristics. Three patients in each group had undergone a colectomy since entering the trial.

The main findings from the patient interviews were that:

- Participants who had received infliximab appreciated the positive outcome from this treatment.
- Those who received infliximab appeared to speak more positively about their treatment than those who received ciclosporin.
- The debilitating symptoms of ulcerative colitis impact not only on their own quality of life, but also on their relationship with family and friends.
- Many participants expressed their desire to return to a 'normal' quality of life and many of those who had a colectomy, whist initially adamant that they did not wish to 'lose' their colon, found relief from surgery and felt they could move on with their lives.
- Patients came to terms with having to live with the ongoing unpredictability of symptoms and treatments yet they recognised that this unpredictability makes it particularly difficult for them and for the healthcare professionals treating them to manage their health.
- Ulcerative colitis is considered an embarrassing condition which makes it an isolating and awkward experience for patients due to its impact on life and work.
- Lack of visibility of either symptoms or outcomes, also affected patients' willingness to share knowledge of the disease with others.
- Surgery was feared, but once a colectomy had been undertaken, most participants experienced relief and recognised the health benefits.

- Participants wanted to know more about the cause of ulcerative colitis and its links with stress and diet, and would have welcomed greater information provision.
- Ready access to an Inflammatory Bowel Disease Specialist Nurse was suggested as particularly important for members of this patient group.

Results – interviews with professionals

Twenty-three interviews were undertaken with 15 principal investigators, stratified by the number of patients recruited per site, and eight senior nurses from sites that recruited well to the trial.

The main findings that emerged from these interviews were that

- Healthcare professionals make judgements about the two drugs largely based on their own personal experience of prescribing or giving them to patients.
- The views of nurses are influenced by the drug therapy choices of their units, the method of administration, and perceptions of effectiveness and adverse side effects.
- A clear preference for infliximab amongst nurses was based predominantly on the ease of administration of a short infusion of infliximab, when compared with the continuous 24 hour infusion required for ciclosporin.
- Some doctors strongly favoured infliximab, wishing to see it as the drug of choice in view of its perceived ability to manage the many complex symptoms of the disease, ease of administration, fewer side effects, and greater effectiveness.
- Other doctors favoured ciclosporin, perceiving it as safe, effective and cheap.
- Most doctors were unsure which drug to use in the future, and were prepared to wait for further evidence of effectiveness and safety before fully making up their minds.
- Doctors questioned guidelines relating to prescribing these drugs, and the restrictions this placed on personal autonomy in delivering best patient care.

Discussion

The number of subjects interviewed in this study was small, but they were representative of the trial participants and sites. The findings include a strong preference from nurses for infliximab, based largely on a dislike of the infusion requirements for ciclosporin. Although doctors were in equipoise with regard to the trial, most but not all of those interviewed wished to see infliximab as the recommended drug of choice in the future. Patients who received infliximab tended to speak more positively their treatment than those given ciclosporin, and those who required surgery were positive about colectomy, having been fearful pre-operatively. The very debilitating and embarrassing impact of the disease on health, work and social life was emphasised by many patients.

References

1. Williams JG, Alam MF, Alrubaiy L, et al. Comparison Of iNfliximab and ciclosporin in STeroid Resistant Ulcerative Colitis: pragmatic randomised Trial and economic evaluation (CONSTRUCT). *Health Technol Assess* 2016; **in press**.
2. Rapport F. Summative Analysis: A Qualitative Method for Social Science and Health Research. *International Journal of Qualitative Methods* 2010; **9**(3): 270-90.
3. Rapport F, Iredale R, Jones W, et al. Decision aids for familial breast cancer: exploring women's views using focus groups. *Health Expect* 2006; **9**(3): 232-44.
4. Ryan GW, Bernard HR. Data analysis and management methods. In: Denzin NK, Lincoln US, eds. Handbook of Qualitative Research. 2nd ed. CA: Sage, Newbury Park, CA; 2000: 769-902.
